# A modified method for molecular identification of *Baylisascaris transfuga* in European brown bears (*Ursus arctos*)

**DOI:** 10.1007/s00436-017-5660-2

**Published:** 2017-10-27

**Authors:** Jakub Gawor, Jan Gawor, Robert Gromadka, Tomasz Zwijacz-Kozica, Filip Zięba

**Affiliations:** 1 Witold Stefański Institute of Parasitology, Polish Academy of Sciences, Twarda 51/55, 00-818 Warsaw, Poland; 2 Laboratory of DNA Sequencing and Oligonucleotide Synthesis, Institute of Biochemistry and Biophysics, Polish Academy of Sciences, Pawińskiego 5a, 02-106 Warsaw, Poland; 3 Tatra National Park, Chałubińskiego 42a, 34-500 Zakopane, Poland

**Keywords:** *Baylisascaris transfuga*, Brown bear, PCR, Chelex resin

## Abstract

*Baylisascaris transfuga* is a roundworm that has been reported worldwide in most bear species. In mammals and possibly humans, the larvae of *B. transfuga* can migrate in the tissues of aberrant hosts with larva migrans syndrome. The current study was performed to identify *B. transfuga* in faecal samples from free-ranging brown bears in the Tatra Mountains National Park in southern Poland. A commercial kit was used to extract genomic DNA directly from faecal samples. Additionally, a Chelex resin-based technique was successfully implemented to prepare a PCR template from eggs retrieved by flotation. Based on the flotation results of 32 collected faecal samples, the prevalence of *B. transfuga* was 15.6%. The parasite was confirmed in samples found to be positive during the initial flotation by a molecular assay using DNA isolated directly from faeces. The retrieved eggs were confirmed as *B. transfuga* after their DNA was extracted using the Chelex protocol. Based on PCR amplification and sequencing of a 413-bp segment of cytochrome c oxidase 1 (COI), the obtained sequence was 100% identical to the COI segment of *B. transfuga* after a BLAST comparison to the GenBank™ database. The current study includes the first molecular confirmation of *B. transfuga* in brown bears in the western part of the Carpathians. We show that direct extraction of parasite DNA from bear faeces is efficient for molecular assays. As an alternative, we present the effectiveness of a Chelex-based technique for fast and convenient DNA isolation from the difficult-to-disrupt eggs of *B. transfuga* for PCR. Molecular tests of parasite DNA extracted directly from faecal material have limits of detection related to the amount of eggs in the samples. Thus, using classical flotation to obtain eggs for PCR may increase the credibility of the results, particularly in cases with a low number of excreted eggs. The Chelex resin protocol has potential for application in studies of intestinal parasites in wildlife for which conventional flotation is routinely used for microscopy.

**Electronic supplementary material:**

The online version of this article (10.1007/s00436-017-5660-2) contains supplementary material, which is available to authorized users.

## Introduction

*Baylisascaris transfuga* is an ascarid nematode species that exclusively infects bears (Bauer 2013). It has been reported worldwide in all extant species of bears in the family Ursidae, excluding the spectacled bear (Sapp et al. 2017). *B. transfuga* occurs in both free-ranging and captive bears, such as the polar bear (Schaul 2006; Testini et al. 2011), American black bear (Duffy et al. 1994; Schaul 2006; Foster et al. 2004; Catalano et al. 2015), grizzly bear (Catalano et al. 2015), sloth bear (Moudgil et al. 2014) and European brown bear (Finnegan 2009; De Ambrogi et al. 2011; Szczepaniak et al. 2012; Bauer 2013; Aghazadeh et al. 2015; Bugmyrin et al. 2017).

Although *B. transfuga* is highly prevalent, as 50–100% of bears have been shown to be infected (Sprent 1968; Catalano et al. 2015), the pathogenicity of intestinal infection appears to be low (Bauer 2013). Nevertheless, for bears kept in captivity, baylisascariasis is a health threat due to contamination of the enclosure environment with infective stages of the parasite. An abundance of hundreds of adult specimens of *B. transfuga* has been reported in two polar bears in a zoo park in Italy (Testini et al. 2011). In the wild, heavy infestations could cause serious illness or death in cubs, as was shown in a young, male European brown bear with extensive abdominal lesions (granulomatous peritonitis) caused by *B. transfuga* (Szczepaniak et al. 2012).

The life cycle of *B. transfuga* is unclear (Bauer 2013), but it may be similar to that of the closely related parasite *B. procyonis*, the raccoon roundworm, a well-known cause of severe to fatal neurological disease in humans and many wildlife species (Sapp et al. 2017). Bears may become infected by ingesting embryonated eggs that are present in the environment. However, it is not known whether under natural conditions the intermediate hosts, which are prey animals, are a source of *B. transfuga* infection for bears (Bauer 2013). As shown in experimental infections, *B. transfuga* larvae are able to migrate in tissues of rodents, i.e. mice and Mongolian gerbils. The larvae migrate through different tissues, grow, and develop to the third stage, causing various degrees of visceral, neural or ocular larva migrans (Papini and Casarosa 1994; Papini et al. 1996; Sato et al. 2005; Cho et al. 2007; Bauer 2013).

To date, there is no unequivocal evidence of naturally occurring *B. transfuga* infection in non-ursid animals or humans (Bauer 2013; Sapp et al. 2017). The only reported case was an outbreak of fatal neurological larva migrans in Japanese macaques (*Macaca fuscata*) that share their living space with American black bears harbouring *B. transfuga*. However, the involvement of this ascarid species was only confirmed by epidemiological observations (Sato et al. 2005).

Fertilized eggs of *B. transfuga* have a thick, mammillated, proteinaceous coat and an impermeable, desiccation-resistant lipid layer (Kazacos and Turek 1983; De Ambrogi et al. 2011). Similar to those of other *Baylisascaris* species, the eggs of *B. transfuga* become infective after approximately 2 weeks and can remain infective for at least 15 months under artificial conditions (Papini and Casarosa 1994). Eggs have been reported to persist under appropriate environmental conditions for up to 5 years (Sapp et al. 2017). Humans might be exposed to a risk of infection in a highly contaminated environment, such as that in animal enclosures in zoo parks and in nature (e.g. zoo staff, trappers and hunters) (De Ambrogi et al. 2011; Testini et al. 2011).

The aim of this study was to evaluate *B. transfuga* infection in bears living in Tatra National Park (the western part of the Carpathians) in southern Poland based on an examination of faecal samples using flotation and molecular identification. We assessed the efficiency of direct isolation of DNA from faecal samples using a commercial kit and a Chelex resin-based method of extracting genomic DNA from eggs isolated after flotation.

## Materials and methods

In the present study, faecal material was collected from brown bears in Tatra National Park (the western part of the Carpathians) in southern Poland (Fig. 1). The protected area covers 212 km^2^ and, according to genetic monitoring (capture-mark-recapture population estimates), is inhabited by 36–52 bears (Zwijacz-Kozica et al. 2014). Because the National Park is visited each year by approximately three million tourists and is surrounded by dense settlements, the appearance of problem bears is a major concern.

### Faecal samples and flotation analysis

Thirty-two faecal samples were collected from brown bears in Tatra National Park. Each faecal sample was recognized as having been deposited by a separate animal. Sampling was performed in 2014 (from March to November) and 2015 (from May to June). Until analysis by flotation and molecular examination were performed, the samples were maintained frozen at −18 °C. To recover *B. transfuga* eggs from bear faeces, flotation with a zinc sulphate (ZnSO_4_)-saturated solution (1.35 specific gravity) was performed (Euzeby 1981). For molecular analysis, the flotation was repeated for each positive sample, and 100 μl of each supernatant with eggs was subjected to a Chelex resin-based DNA extraction (Walsh et al. 1991). Five faecal samples that were positive for *B. transfuga* eggs and five samples that were negative for eggs at the time of flotation analysis were used for molecular testing by PCR, with genomic DNA isolated directly from faecal samples using a commercial kit.

### Molecular procedures

#### Genomic DNA isolation

Extraction of genomic DNA consisted of two parts:

- Direct isolation of genomic DNA from faecal samples.
  In all, 200 mg of each sample was weighed for DNA extraction using a soil DNA extraction kit (Eurx, Poland) according to the manufacturer’s instructions.
- Chelex resin-based DNA preparation (Walsh et al. 1991) from *B. transfuga* eggs recovered by flotation.

*B. transfuga* eggs were washed three times with MilliQ water to remove excess salts after the flotation procedure, and at each stage, centrifugation at 14000 rpm was performed for 5 min to pellet the eggs prior to supernatant removal. Eggs were then re-suspended in 100 μl of MilliQ water. Twenty microlitres of the suspension was added to a 10% Chelex resin solution (1 g of Chelex® 100 Resin (Sigma) in 10 ml of MilliQ water). Approximately 100 μl of 0.5 mm diameter glass beads (Sartorius AG, Germany) were added to the sample, and the probes were pulverised for 10 min at 20 Hz in a TissueLyser II homogeniser (Qiagen, Germany) to mechanically disrupt the nematode eggs. After homogenization, 3 μl of proteinase K (20 mg/ml, Sigma, USA) was added, and the sample was incubated at 55°C for 30 min, followed by 5 min of incubation at 95°C to inactivate the enzyme. Finally, the sample was centrifuged at 14000 rpm for 5 min, and the supernatant was transferred into a new Eppendorf tube.

### PCR primers

To amplify a 413-bp segment of cytochrome c oxidase 1 (COI), the COI-F and COI-R primers were used according to Sato et al. (2005).

### COI amplification by PCR

PCR was carried out in 20 μl volumes using OptiTaq polymerase (Eurx, Poland) and 2 μl of DNA. The reaction conditions were as follows: initial denaturation at 94°C for 3 min followed by 35 cycles of denaturation at 94°C for 15 s, annealing at 50^°^C for 15 s and elongation at 72^°^C for 1 min. Final elongation was performed at 72 °C for 2 min. The resulting PCR products were visualized on 1% agarose gels and purified using an exonuclease I/alkaline phosphatase mix (Thermo Scientific, USA). Positive samples were sequenced with PCR primers using BigDye Terminator v. 3.1 chemistry and an ABI3730xl genetic analyser at the DNA Sequencing Laboratory (Institute of Biochemistry and Biophysics, Polish Academy of Sciences). Sequencing reads were assembled into contigs using Seqman software (DNAStar, USA). The consensus nucleotide sequence of the COI fragment was aligned to the reference sequence obtained from the GenBank™ database (Johnson et al. 2008) using BLAST (Altschul et al. 1990).

## Results

### Flotation analysis

Based on the initial flotation analysis, the prevalence of ascarids in bears was 15.6%. Among the 32 faecal samples that were analysed, five contained 31, 97, 105, 116 or 241 eggs per 1 g of faeces.

### PCR

After confirmation of *B. transfuga* eggs in five samples by initial flotation analysis, DNA was directly isolated from each positive faecal sample using a commercial kit. All of these samples were found to be positive by using amplification with specific primers, that is., a 420-bp COI fragment was generated by PCR. The five samples that were negative for eggs in flotation analysis were tested in the same way, and one of them was identified as positive by the molecular assay.

Five supernatants with eggs from the positive samples recovered by flotation were subjected to a manual DNA isolation procedure using Chelex resin. PCR amplification was successful for all five of the tested samples. During the PCR amplification of COI, faeces with no eggs and a water sample were used as negative controls. The *B. transfuga* COI-specific PCR product from one positive sample isolated by Chelex resin was sequenced. The resulting sequence of the fragment was a 100% match to the *B. transfuga* mitochondrial COI sequence (accession number HQ671079) based on a BLAST comparison to the GenBank™ database. The nucleotide sequence was deposited in the GenBank™ database under the accession number KY973960.

## Discussion

The current study reports molecular identification of *B. transfuga* in the small population of free-living brown bears in the protected area of Tatra Mountains National Park. Genomic DNA of the parasite was obtained from eggs retrieved from faecal samples after preliminary flotation by direct extraction from the same samples using a commercial kit. Extraction of DNA from ascarid eggs for PCR requires mechanical damage of their sheath (Dangoudoubiyam et al. 2009; De Ambrogi et al. 2011) or the use of enzymatic lysis (proteinase K) for an extended period of time. Lysis was previously used to obtain genomic DNA from soil samples contaminated with *Toxocara* spp. eggs (Borecka and Gawor 2008). De Ambrogi et al. (2011) successfully performed mechanical disruption of *Baylisascaris transfuga* eggs using a mixer mill with one stainless steel bead per sample, developing a method to extract DNA and run PCR directly from faecal samples. We directly detected the DNA of the parasite in bear faecal samples using a commercial kit, which was confirmed by amplifying the *cox*1 gene fragment of the *B. transfuga* mitochondrial genome. PCR testing with directly extracted DNA was sensitive, as one positive sample was revealed among the five samples that were initially identified as negative during the initial flotation analysis.

A Chelex-based method (Walsh et al. 1991) was successfully adapted to prepare a PCR template from *B. transfuga* eggs. The samples were vortexed with 0.5 mm glass beads to mechanically disrupt the eggs in the presence of chelating Chelex resin, and proteinase K was then added for additional digestion of the egg coat. Chelex 100 resin is a chelating resin that enables ion exchange to bind transition metal ions. During the extraction process, the alkalinity of the solution and boiling break down cells and allow the chelating groups to bind to the cellular components, which protects DNA from degradation (Phillips et al. 2012). To our knowledge, Chelex resin has not been used previously to extract DNA from any ascaridoid nematodes.

Our attempts to perform Chelex resin extraction from regular faeces failed. The lack of a PCR amplification product was probably due to the residual PCR polymerase inhibitors that commonly exist in faeces.

Diagnosis of a *B. transfuga* infestation in bears commonly relies on the retrieval of eggs by conventional faecal flotation analysis (Finnegan 2009; De Ambrogi et al. 2011; Catalano et al. 2015; Bugmyrin et al. 2017). The eggs can be easily recognised; therefore, detection of the eggs by microscopy after flotation seems to be easy for the observer, except that the eggs of *B. transfuga* cannot be distinguished from those of other ascarids (Kazacos and Turek 1983). The sensitivity of faecal flotation techniques may be limited for *B. transfuga*, particularly due to its long prepatent period, negative results with even a high-intensity intestinal infection (Testini et al. 2011) and seasonal trends in the prevalence of *B. transfuga* in bears (Bugmyrin et al. 2017; Sapp et al. 2017). De Ambrogi et al. (2011) showed that the sensitivity of flotation for *B. transfuga* eggs was 60%, with mixed results when flotation was repeated several times with the same samples. Unfortunately, the authors did not mention the number of eggs per gram that was retrieved by flotation from the collected faecal samples.

Different procedures have been used to test the capacity of a given method to detect DNA in *Baylisascaris* eggs, i.e. uninfected faecal samples are spiked with eggs and flotation is performed to retrieve the same eggs (Dangoudoubiyam et al. 2009) or samples spiked with the eggs are immediately subjected to extraction (De Ambrogi et al. 2011). In the present study, all five of the positive samples were positive according to the PCR assay with DNA isolated directly from faecal material. Moreover, one sample that scored negative in the preliminary flotation analysis was found to be positive in PCR analysis. De Ambrogi et al. (2011) demonstrated limited detection by PCR for 0.025 g aliquots of faecal matter spiked with two *B. transfuga* eggs, and no band was detected in PCR products from samples spiked with a single egg. These results show that a minimum of 80 eggs per gram of faeces is required to confirm infection by molecular examination. The present study shows that the sensitivity of molecular detection is related to the number of eggs recovered by flotation. Our results showing the high sensitivity of direct extraction of DNA from bear faecal samples combined with a PCR assay to obtain a 420-bp product with amplified COI fragments are in agreement with those of De Ambrogi et al. (2011).

This study presents the first screening of *B. transfuga* based on faecal sample examination with molecular identification via a PCR assay in the brown bear population in Tatra National Park in southern Poland. *B. transfuga* was first identified in a brown bear cub that was found dead in the Tatra Mountains in Poland; the cause of death was determined to be granulomatous peritonitis due to extra-intestinal ascariasis via morphological identification at necropsy (Szczepaniak et al. 2012). In Europe, *B. transfuga* has been reported in free-living populations of brown bears in Slovakia (Finnegan 2009) and Croatia (De Ambrogi et al. 2011), with prevalence of 47 and 13.5%, respectively, according to flotation results. A recent survey in the eastern part of the Scandinavian Peninsula (the Kola Peninsula, Russia) revealed *Baylisascaris* sp. eggs in 37.6% of brown bears (Bugmyrin et al. 2017). In the studies of Finnegan (2009) and Bugmyrin et al. (2017), the eggs detected in the samples were not identified by molecular assays and therefore probably belong to *Baylisascaris transfuga*. A study in Canada (Alberta and British Columbia) found the total prevalence of *B. transfuga* in the intestine of American black bears to be 60% (with 42.9% in cubs, 73.7% in juveniles and 50% in adults), while the prevalence was 53.8% in juvenile and adult grizzly bears (Catalano et al. 2015).

Infected bears can pass between 100 and 19,800 eggs per gram of faeces, and thus, environments can quickly become contaminated with large numbers of eggs. *Baylisascaris* eggs that are present in the environment or in captive animal facilities are difficult to eliminate or kill (Sapp et al. 2017). In Tatra National Park, food-conditioned brown bears approach tourist facilities inside the park and often enter neighbouring settlements; therefore, the possible risk to humans cannot be ignored. While there are no confirmed reports of larva migrans in humans following *B. transfuga* infection, experimental evidence with other *Baylisascaris* species shows that given a sufficiently high infection rate, larva migrans in people may be possible (Sapp et al. 2017).

In this study, faecal samples were collected from May to October. Due to the low number of secured samples, it was impossible to assess the seasonality of egg excretion. The prevalence of *Baylisascaris* eggs in faecal samples demonstrates a clear seasonal pattern, with peaks in summer (Bugmyrin et al. 2017). The large, three-year study of Finnegan (2009) in the Carpathian Mountains with 188 faecal secured samples showed seasonal changes in the prevalence of *Baylisascaris* sp. eggs in faeces: 9.5% recorded in spring, 44.6% in summer and 70.8% in autumn. These data indicate that faecal sampling in fall allows one to obtain a more accurate picture of bear infection in a given area. However, some studies attempting to investigate seasonal trends of the prevalence of *B. transfuga* have shown conflicting results, with a higher prevalence in spring compared to fall or the opposite seasonal trend with peaks in fall (Sapp et al. 2017).

## Conclusions

Diagnosis of *B. transfuga* in bears based on the examination of faecal samples requires molecular identification of genetic material isolated from excreted eggs. We show that direct extraction of *B. transfuga* DNA from bear faeces using a commercial kit is efficient for molecular assays. As an alternative, we present the effectiveness of a Chelex-based technique for extracting DNA from eggs. Although the proposed Chelex protocol has limitations related to the need to recover eggs from faecal samples, it allows easy and fast DNA isolation from the difficult-to-disrupt eggs of *B. transfuga*. Molecular tests including DNA of parasites extracted directly from faecal material have limits of detection related to the amount of eggs in the samples. Thus, using classical flotation to obtain eggs for PCR may increase the credibility of results, particularly in cases with a low number of excreted eggs. Microscopy remains the cornerstone for the diagnosis of many parasites, particularly for the detection of rare or emerging parasitic infections in humans and animals. The proposed Chelex protocol has a potential application in studies on intestinal parasites in wildlife in which conventional flotation is routinely used for microscopy.

## Electronic supplementary material

Fig. 1. Study area: Tatra National Park, Western Carpathians, Poland (JPEG 472 kb)
