# Supplementary figures and images for: A modified method for molecular identification of Baylisascaris transfuga in European brown bears (Ursus arctos)
Source: Parasitol Res. 2017 Oct 27;116(12):3447–52. doi: 10.1007/s00436-017-5660-2 (PMC5691110; doi:10.1007/s00436-017-5660-2)

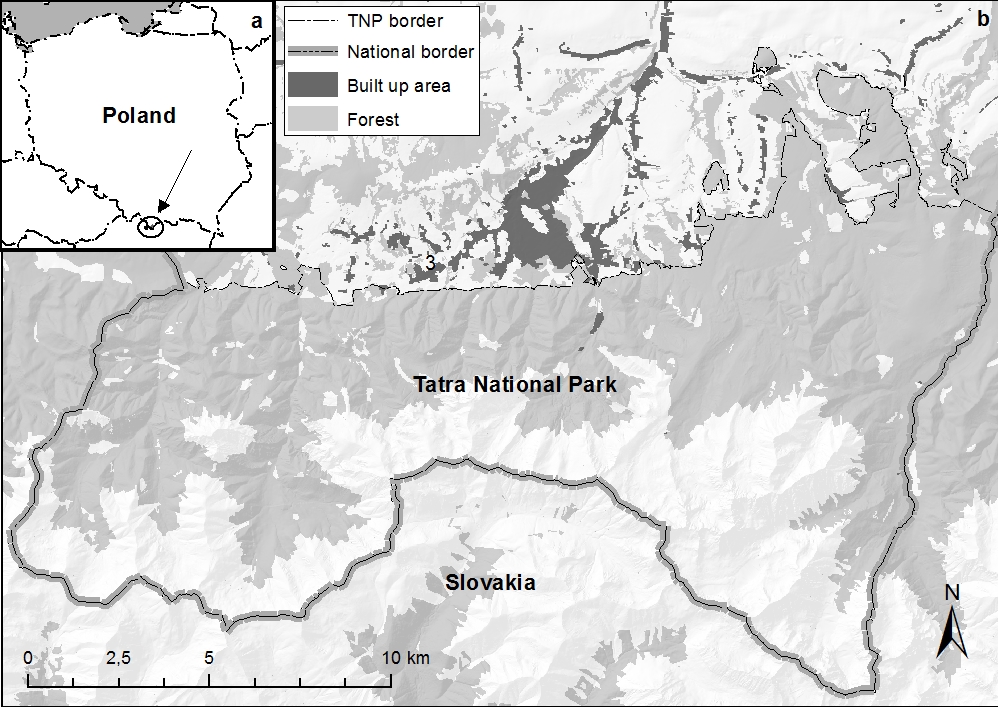

Supplement: Supplementary file 1 — Study area: Tatra National Park, Western Carpathians, Poland (JPEG 472 kb) [file 436_2017_5660_MOESM1_ESM.jpg]
